# Advances in clinical pharmacy education in Germany: a quasi-experimental single-blinded study to evaluate a patient-centred clinical pharmacy course in psychiatry

**DOI:** 10.1186/s12909-017-1092-z

**Published:** 2017-12-12

**Authors:** Monika Dircks, Andreas Mayr, Annette Freidank, Johannes Kornhuber, Frank Dörje, Kristina Friedland

**Affiliations:** 1 Pharmacy Department, Erlangen University Hospital, Erlangen, Germany; 2 Department of Medical Informatics, Biometry and Epidemiology, Friedrich-Alexander University Erlangen-Nuremberg, Erlangen, Germany; 3 Department of Pharmacy, Fulda Hospital, Fulda, Germany; 4 Department of Psychiatry and Psychotherapy, Erlangen University Hospital, Erlangen, Germany; 5 Molecular and Clinical Pharmacy, Friedrich-Alexander University Erlangen-Nuremberg, Erlangen, Germany

**Keywords:** Pharmacy education, Clinical pharmacy course, Teaching and learning study

## Abstract

**Background:**

The pharmacy profession has shifted towards patient-centred care. To meet the new challenges it is necessary to provide students with clinical competencies. A quasi-experimental single-blinded teaching and learning study was carried out using a parallel-group design to evaluate systematically the benefits of clinical teaching in pharmacy education in Germany.

**Methods:**

A clinical pharmacy course on a psychiatric ward was developed and implemented for small student groups. The learning aims included: the improvement of patient and interdisciplinary communication skills and the identification and management of pharmaceutical care issues. The control group participated only in the preparation lecture, while the intervention group took part in the complete course. The effects were assessed by an objective structured clinical examination (OSCE) and a student satisfaction survey.

**Results:**

The intervention group achieved significantly better overall results on the OSCE assessment (46.20 ± 10.01 vs. 26.58 ± 12.91 of a maximum of 90 points; *p* < 0.0001).The practical tasks had the greatest effect, as reflected in the outcomes of tasks 1–5 (34.94 ± 9.60 vs. 18.63 ± 10.24 of a maximum of 60 points; p < 0.0001). Students’ performance on the theoretical tasks (tasks 6–10) was improved but unsatisfying in both groups considering the maximum score (11.50 ± 4.75 vs. 7.50 ± 4.00 of a maximum of 30 points; p < 0.0001). Of the students, 93% rated the course as practice-orientated, and 90% felt better prepared for patient contact. Many students suggested a permanent implementation and an extension of the course.

**Conclusions:**

The results suggest that the developed ward-based course provided learning benefits for clinical skills. Students’ perception of the course was positive. Implementation into the regular clinical pharmacy curriculum is therefore advisable.

**Electronic supplementary material:**

The online version of this article (10.1186/s12909-017-1092-z) contains supplementary material, which is available to authorized users.

## Background

The pharmaceutical profession has rapidly changed in Germany: pharmacists increasingly provide patient-centred care beyond the traditional medication-dispensing role in both ambulatory and hospital care. Various projects in pharmacist-delivered medication services have been successfully undertaken in community settings [1–4]. In several hospitals, pharmacists play an important role on interdisciplinary teams providing direct patient care: they participate in ward rounds, take medication histories, conduct medication reviews and provide discharge counselling [5–8]. The change towards patient-centred care is also made apparent by the update of the Ordinance on the Operation of Pharmacies in 2012 that specifies medication therapy management as a pharmaceutical task [9]. The recently developed “Pharmacy 2030” vision statement of the Federal Union of German Associations of Pharmacists (ABDA) includes not only the traditional dispensing role of a pharmacist but also the relatively new concept in Germany of the joint responsibility of medical doctors and pharmacists for patient medication therapy [10]. Recently, the ABDA also published a position paper about medication regimen review and medication management [11]. However, the low ratio of hospital pharmacists - 0.37 pharmacists/100 patient beds - and the lack of remuneration for patient-centred care in community pharmacies clearly show that Germany still has a long way to go until high-quality clinical pharmacy services become a widespread standard in health care [12].

As a result of these ongoing changes pharmacists are increasingly faced with new roles and duties. To enable the next generation of pharmacists to meet these new challenges and allow them to drive the profession actively forward, new clinical competencies and skills must be taught using new teaching methods in German clinical pharmacy academic education.

Traditionally, the pharmacy education in German universities focused on drugs themselves and focused less on clinical use and direct patient care. This changed in 2001, when the new subject “Clinical Pharmacy” was officially implemented into the nationwide pharmacy curriculum to adjust learning content to the patient-orientated clinical skills needed in practice. However, the proportion of patient-orientated teaching and learning subjects represent only 15% of the entire curriculum, which compromises clinical pharmacy, physiology, pathophysiology, pathobiochemistry, pharmacology, toxicology and anatomy, based on the pharmacy study regulations at the Friedrich-Alexander University Nuremberg-Erlangen [13].

Aiming for the most effective use of teaching time, the “Clinical Pharmacy” working group of the German Pharmaceutical Society (DPhG) published 10 statements in 2004 including the following: patient notes and data should play a central role; direct patient contact is desirable; clinical working pharmacists and physician should be involved in teaching; and to ensure case-based and interactive learning, small learning groups should be formed [14]. The stated aim is to enable students to support patients and medical staff in order to ensure the best possible medication use, taking into account patients’ medical history and current medication use. In countries such as the United Kingdom (UK) or the United States of America (USA), teacher practitioner/preceptors are well-established positions in pharmacy education [15, 16]. They facilitate the contact between faculty and the actual work environment and aid the development of skills such as problem solving, communication, application of knowledge, information retrieval and professionalism [17]. The benefit of clinical education has also been shown in other clinical disciplines, such as medicine and nursing [18–21]. Despite its positive track record, clinical education and direct patient contact within the German pharmacy education is very limited. A pilot project was conducted in 2002/2003 at the University of Bonn, and in some universities motivated hospital pharmacists voluntarily offer ward-based internships for a limited number of students [22, 23]. These activities are very valuable efforts, but clinical education courses have not been regularly implemented within the German pharmacy curriculum due to monetary and time restrictions.

Therefore, a bed-side education course led by a ward pharmacist (teacher practitioner, TP) was developed, implemented and systematically evaluated to demonstrate the benefits of such a course in the German setting. The main learning aims of the course were (i) improving patient communication skills and (ii) learning and practising clinical skills to be able to identify and solve pharmaceutical care issues. The teaching project was externally funded by a distinguished pharmaceutical foundation and lasted 3 years. The evaluation took place in the second year of the project. Participation in the course and its evaluation was voluntary for the students. This article presents the structure of the bed-side pharmacy course carried out on a psychiatric ward of a German university hospital and its systematic evaluation.

## Methods

Professional competencies that enable pharmacy students to provide patient-centred care cannot be sufficiently taught in a classroom environment. Therefore, a clinical pharmacy course in a clinical setting was developed. Case-based learning was integrated as an active-learning style to support self-learning skills [24]. To support cooperation and communication, to stimulate critical thinking and to encourage discussions, team–based learning in small groups was the chosen teaching strategy, as described elsewhere [25].

Learning objectives included increasing knowledge about applied drug therapy, collecting patient-specific data, assessing the drug regimen of patients considering comorbidities and laboratory tests, identifying drug related problems and prioritizing them based on urgency and severity, counselling patients on drug therapy given patient-specific factors, and improving communication skills with both patients and medical staff.

The TP was fully integrated into the ward team participating regularly in ward rounds, taking drug histories and counselling patients regarding their drug regimen. Fifty percent of the TP’s work time was dedicated solely to teaching activities. The project was conducted in a friendly and highly supportive work environment, comprising the faculty of Clinical Pharmacy, the Psychiatric Clinic and the Pharmacy Department of the tertiary care University Hospital Erlangen.

The core element of the new course was a patient-centred ward-based placement (2 × 4 h) supervised by the TP. For preparation, a lecture (1.5 h) and a seminar (1.5 h) were held in the classroom in advance (Table 1).

**Table 1:** Learning content of the course modules

Course module	Learning content
Preparation lecture (1.5 h)	Definition of medication therapy management and drug therapy review Tools for a drug therapy review - SOAP (subjective and objective patient data, assessment: identification of drug-related problems, plan: handling of drug-related problems) - Medication Appropriateness Index [26] Example of a drug therapy review (presentation) Introduction to the psychiatric ward
Preparation seminar (1.5 h)	Communication with (psychiatric) patients Pharmaceutical problems on psychiatric wards Drug therapy review performed in small student groups (paper case)
Ward-based placement – Day 1 (4 h)	Drug therapy review on the ward including taking a drug history - Gathering and assessing relevant pharmaceutical information - Performing a patient interview - Identification and handling of drug-related problems including communication with the responsible prescriber
Ward-based placement – Day 2 (4 h)	Counselling of a psychiatric patient regarding drug therapy, including - Important counselling topics, e.g., adherence - Individualization and prioritization of content

Due to the high prevalence of psychiatric diseases and to improve the understanding of psychiatric illnesses and their associated problems the course was held on an acute psychiatric ward. The strong interdisciplinary relationship between the pharmacy department and the psychiatric clinic was an additional reason for conducting the course in this setting. Typical illnesses of the patients participating in the course included depression, schizophrenia and bipolar disease and patients were asked for their consent. The course required students with knowledge in psychiatric and general pharmacotherapy; it therefore included students in their final (8th) school semester. Modifications were made to the clinical pharmacy lectures held at the university in the previous year. All students had to pass an oral exam assessing their clinical knowledge before they were allowed to participate in the study. Students were allowed to repeat the examination twice. All students passed the exam.

The preparation lecture was held at the university. The students were provided basic information, including an introduction to the psychiatric ward, and explanation of the role of the ward pharmacist, how to take medication histories and how to perform a medication therapy review. An introduction to SOAP (subjective, objective, assessment, plan) notes and the Medication Appropriateness Index (MAI) was given [26]. Finally, a medication therapy review of a psychiatric patient was presented.

The preparation seminar employed active-learning methods. Every team consisted of 3 students and was asked to design a patient care plan. For this task, students needed to identify patient data from medical notes and perform a medication therapy review to identify drug-related problems. They had to apply methods for providing patient care learned in the preparation lecture and their prior studied knowledge of pharmacotherapy. The results were discussed collectively at the end of the seminar, and a care plan was created.

The first day of the clinical course concentrated on taking medication history and identifying and handling drug-related problems. The students screened the medical notes independently to identify relevant patient data and were expected to perform a patient interview focusing on drug treatment. The interviews were carried out on the ward in a confidential environment (e.g., single patient room or doctor office). The TP supervised the interviews at all times. To enhance students’ understanding of psychiatric patients and their specific problems the patients were also asked to describe their experience with the disease. The content of the interview and communication techniques, such as active listening, open-ended questions and non-verbal communication, were discussed in advance. For most students, this was their first direct patient contact. After the completion of the patient interview, informal feedback was given to the individual student by the group and the TP. For the assessment of the drug treatment, the information gained in the interview and the medical records, including laboratory data were used. Any problems found should have been discussed with the responsible prescriber. Due to time restrictions of the medical staff, this was done with the TP acting as the physician.

On the second day of the course, the focus was on counselling psychiatric patients on their drug regime. The students thoroughly prepared their educational talk and discussed important issues with the TP, including adverse effects, possible interactions with other drugs or food, duration of treatment and delay in the onset of the effects of the medication. Since psychiatric drug therapy, its adverse effects and adherence are demanding topics, the TP performed counselling as the students observed. A thorough discussion followed about the communication technique and talk structure used.

The exercises in the clinical pharmacy course mostly engaged the lower and middle level of learning within Bloom’s revised taxonomy (remembering, understanding and applying). Some higher-level (analysing) learning occurred [27].

The clinical pharmacy course was evaluated using an objective structured clinical examination (OSCE). In addition, a student satisfaction survey was administered to gauge students’ opinions about the course structure, the relevance of teaching content and their overall satisfaction.

### Objective structured clinical examination

Common examination methods used in pharmacy education in Germany are written and oral examinations that test mainly for factual knowledge. In the evaluation of the clinical education course, both communication skills and the application of clinical knowledge needed to be assessed. Therefore, the traditional examination methods of oral and written examination or multiple choice tests were deemed unsuitable for our investigation, and OSCE was chosen instead. Since its introduction in the 1970s by Harden, OSCE has become a well-established tool for assessing clinical competence [28]. OSCE typically consists of different tasks involving a patient actor as a “standardized patient” and an observing evaluator. This allows the assessment of clinical skills such as clinical knowledge, professional judgment, communication, interpersonal skills, problem-solving skills, and resolution development [29]. OSCEs have been shown to be valid and reliable in assessing clinical competence in pharmacy and other health care professions [30–32]. However, OSCE is not regularly used as an examination method in German pharmacy education.

**Fig. 1:**
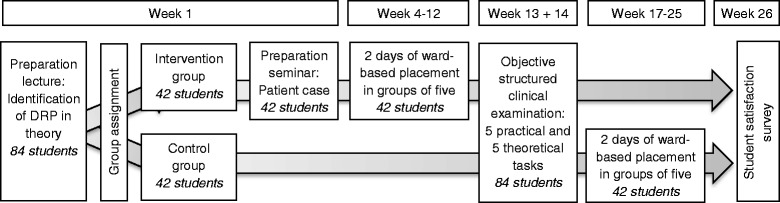
Study design

We undertook OSCE as a single-blinded quasi-experimental controlled trial with a parallel-group design in the second year of the project. All students in this class (*n* = 84) were asked to choose a placement date and were thus unknowingly separated into 2 equal-sized groups. The intervention group completed the entire course; the control group took part only in the preparation lecture (Fig. 1). The latter took part in the ward-based course after the examination. During the OSCE assessment, 10 tasks with psychiatric and non-psychiatric issues were given to all students, similar to circuit training. These tasks were separated into 5 theoretical and 5 practical tasks (Table 2). In each theoretical task, the students had to identify drug-related problems (e.g., an unnecessary duplication or adverse effects) in a paper case. In the practical tasks the students had to solve a given task while interacting with an actor playing a patient or a physician, e.g., patient counselling or informing an uncooperative physician about adverse effects and the necessary therapy modifications. Performance in hard skills (factual knowledge) and soft skills (communication) was assessed by an additional observing person using an evaluation check list. The actor and the additional person were pharmacists trained beforehand for their role in the examination to ensure a standardized assessment. Both were blinded in regard to the intervention or control group assignment of the students. The tasks were developed by the TP based on typical problems pharmacists face in clinical practice. To ensure a realistic level of difficulty the tasks were pretested with preregistration pharmacists who attended comparable clinical pharmacy lectures at the same university.

**Table 2:** Tasks in the Objective Structured Clinical Examination (OSCE)

Task No.	Practical OSCE tasks
1	Counselling of a depressive patient with a newly prescribed neuroleptic drug for psychotic symptoms.
2	Communication with an uncooperative doctor about necessary interventions in a surgical patient with lithium intoxication.
3	Identification of drug-related problems when interviewing a patient for a drug history.
4	Counselling of a patient who had recently started taking antihypertensive medication.
5	Counselling of a non-adherent patient regarding antidepressant medication.
	Theoretical OSCE tasks
6	Identification of drug-related problems in a schizophrenic patient with metabolic syndrome who is supposed to be starting on quetiapine.
7	Identification of drug-related problems: diagnosis without mandatory medication and a drug without indication.
8	Identification of drug-related problems: a review of the medication in consideration of blood values (hyperkalaemia).
9	Identification of drug-related problems while changing out-patient medication to drugs stocked in the hospital pharmacy (drug formulary) at admission.
10	Identification of drug-related problems: a review of the medication in consideration of blood values (QT prolongation and hypokalaemia).

The OSCE results are reported as the median and interquartile ranges (IQR). Group differences were assessed via the non-parametric Wilcoxon signed rank test while adjusting for multiple testing via the Benjamini-Hochberg method.

### Student satisfaction survey

For further evaluation of the pharmacy course a student survey was performed after all students completed the course to learn students’ perception. The survey was developed based on questionnaires previously used at the university. The 22 questions contained general questions, such as prior interest in the course and overall satisfaction, and more specific questions, such as how the topics related to practice as well as the structure of the course. Open questions invited the students to state their general impressions and ideas for improvements.

## Results

### OSCE

**Fig. 2:**
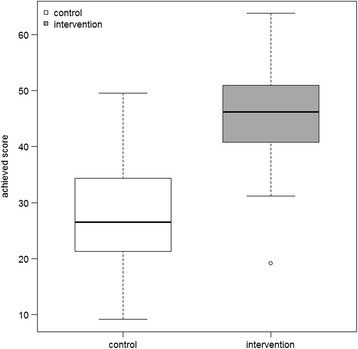
Boxplot representing the empirical distribution of achieved total scores from both groups

**Fig. 3:**
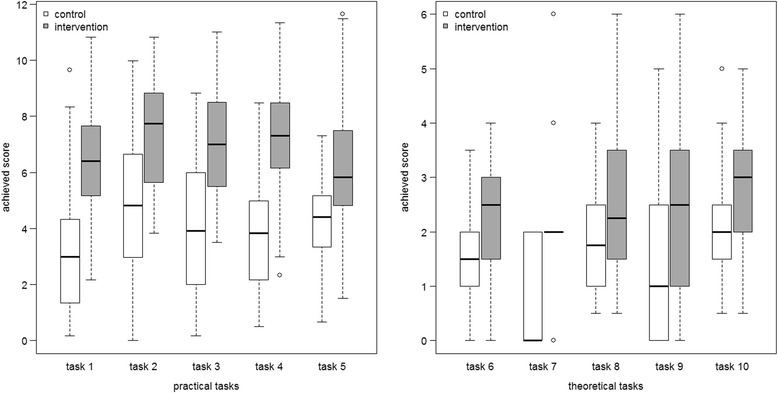
Boxplot representing the empirical distribution of achieved scores in individual tasks

The intervention group achieved significantly better overall results on the OSCE assessment (46.20 ± 10.01 vs. 26.58 ± 12.91 of a maximum of 90 points; *p* < 0.0001) (Fig. 2). The largest effect was in the practical tasks, as reflected in the outcome of tasks 1–5 (34.94 ± 9.60 vs. 18.63 ± 10.24 of a maximum of 60 points; *p* < 0.0001) (Fig. 3). The performance in the theoretical tasks (tasks 6–10) was improved but unsatisfying in both groups considering the maximum score (11.50 ± 4.75 vs. 7.50 ± 4.00 of a maximum of 30 points, *p* < 0.0001). To further analyse these results, the practical tasks were divided into hard skills (factual knowledge) and soft skills (communication skills). The intervention group achieved significantly higher scores than the control group in the latter (27.28 ± 8.34 vs. 16.00 ± 9.07 of a maximum of 40 points; p < 0.0001), while the effect in hard skills was smaller (6.73 ± 3.95 vs. 3.83 ± 2.79 of a maximum of 20 points; p < 0.0001).

Four students in the intervention group and 5 students in the control group completed vocational training to be a pharmacy technician before they entered university education. No influence of this prior knowledge was detected via statistical regression analysis (results not shown). Raw data of the OSCE assessment are provided in Additional file 1.

### Student satisfaction survey

**Fig. 4:**
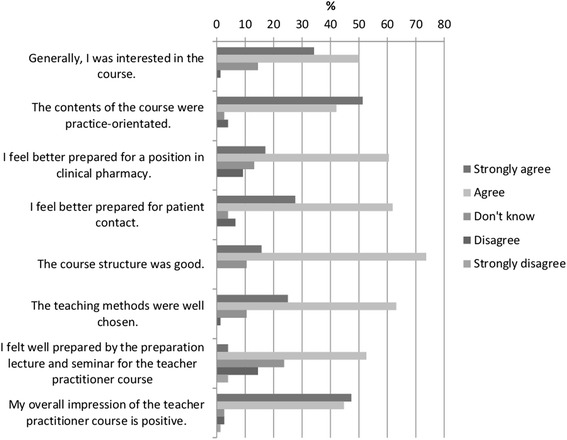
Student satisfaction survey. 76 (=100%) out of 84 surveys were returned

Seventy-six of 84 (90%) students completed the survey. The feedback reflected a very positive reception of the course, as shown by the example questions in Fig. 4. Of the students, 93% rated the course as practice-orientated, 90% felt better prepared for patient contact, and 92% gave a positive answer about their overall satisfaction.

The predominantly positive answers to the open questions support these results. Many students suggested a permanent implementation of clinical education in the pharmacy course curriculum; others suggested an extension of the course length and the inclusion of different medical fields, such as cardiology or endocrinology. Examples are shown in Table 3.

**Table 3:** Example answers of open questions in the student satisfaction survey (translated)

Definitely one of the best courses in our pharmacy studies. It should be extended.
More courses such as this (practical topics, small student groups) should be offered.
The following topics were very important and useful: problems in the patient interview and patient counselling, explaining important issues in an easy way for the patient and improving compliance.
I found it very useful to learn in the real world how to deal with difficult [psychiatric] patients instead of learning about theoretical patient cases.
The project should definitely be continued!
This course should be offered to all pharmacy students in Germany.

## Discussion

For the first time, a systematic investigation of a ward-based clinical education course fully integrated into the curriculum was conducted in German pharmaceutical education. Previous courses were either not offered for all students or not evaluated using parallel-group design [22, 23]. The participation of all 4th-year pharmacy students during all 3 consecutive years was voluntary, but all students took the opportunity to participate in this learning course. Their learning success was thoroughly examined employing an OSCE evaluation and assessment techniques [28, 29]. Students’ performance in the course and examination did not influence their grades. Faculty members responsible for final examinations in clinical pharmacy were blinded to the OSCE results. Students’ perception of the ward-based clinical pharmacy course was further assessed by a comprehensive student satisfaction survey. The design of an OSCE circuit with various exercises and different evaluators improved the reliability of the examination. Subjectivity bias was avoided through the use of blinded external observer and evaluation check lists.

Despite the high workload in the final year of their education, the students participated very actively in the course. Their positive learning attitude and high motivation were also reflected in the high number of very positive answers in the survey. Students greatly appreciated patient contact, the opportunity to gain insight into a clinical work environment and the opportunity to apply their knowledge in practice.

Overall, the OSCE results suggested a positive effect of the clinical pharmacy course on students’ performance. Considerable improvements were achieved in the practical tasks, while the effect in the theoretical tasks was smaller. This difference can be explained by distinguishing hard skills (factual knowledge) from soft skills (communication skills). The results suggested that the course benefitted communication skills, as students improved significantly (27.28 ± 8.34 vs. 16.00 ± 9.07 of a maximum of 40 marks; *p* < 0.0001). However, the OSCE results in the theoretical tasks that showed the ability to apply clinical knowledge were rather disappointing, considering the students were in their final semester (11.50 ± 4.75 vs. 7.50 ± 4.00 of a maximum of 30 marks; p < 0.0001). The wide variability in the theoretical results might show that these tasks heavily depend on the individual students’ preparation. This preparation was highly variable since the OSCE results did not influence any students’ grades and the assessment was carried out anonymously. Furthermore, students’ multiple official learning and examination obligations in the context of the current pharmacy educational curriculum within the final educational year were very demanding. This might also have contributed to a variable preparation for the OSCE. Given the fact that the students overall exposure to the clinical setting and direct patient care experience was limited to 8 h, the assessment results reflected a realistic examination expectation. The OSCE is not used in German pharmacy education and the stress of a new examination technique might have hampered students’ performance. In further studies students should be prepared by practising the OSCE in advance. Another factor contributing for the poor results could have been the small proportion of curriculum hours appointed for clinical pharmacy. This might have simply been insufficient for a thorough clinical education. The small effects of the course on factual knowledge could have also been caused by the variable learning and teaching content on the individual day, which depended heavily on the participating patient.

The participation of physicians in the clinical course was very limited despite the very positive attitude towards the bed-side teaching course. Time restrictions of the medical staff made it difficult for the students to discuss identified problems with them and thus improve their inter-professional communication skills. Instead, the students discussed the issues with the TP, who acted as a physician. However, in OSCE task 2, the intervention group demonstrated better communication skills with an uncooperative doctor than the control group did.

In Germany, the implementation of ward-based clinical education into the systematic teaching and learning of clinical pharmacy is hampered due to financial restrictions. Ward-based teaching can be performed only in small groups with close supervision, making the course very time-consuming. Pharmacists who work regularly on wards and are competent in teaching are needed as tutors. This requirement currently cannot be met by the majority of German universities. Currently, no German pharmaceutical educational curriculum is implemented that recognizes future needs to systematically teach and learn in a ward-based clinical setting while embracing the patients care experience. TP positions are not established on a regular basis, as for example, in the UK or the USA. The guidance of the American College of Clinical Pharmacy for the interpretation of the Accreditation Council for Pharmacy Education standards clearly shows the discrepancy in the different standards between the USA and Germany. The USA requirements are that introductory pharmacy practice experiences should encompass at least 5% of curricular content or 300 h, whereas there are no such requirements at all in the German pharmacy course curriculum [33].

Beyond the benefits shown in this study students may have developed on a personal level, which is difficult to test in an OSCE. Discussions and questions asked by the students during the course and thereafter suggested a learning benefit in terms of attitude towards mental health patients. This impression was supported by previous research showing that contact-based education was an effective method for reducing mental illness-related stigma among pharmacy students [34].

To generalize the results of this study to German pharmacy education, further studies involving multiple study sites in Germany must be carried out. Nevertheless, the results of this study supported by the experience of ward-based courses led by a teacher practitioner in other countries and other disciplines [18–21] strongly suggest that this teaching module should become a fully integrated standard of teaching and learning in future clinical pharmaceutical education in Germany.

## Conclusions

To meet the new challenges arising in the pharmacy profession, pharmacists must be taught clinical skills such as application of clinical knowledge and communication skills.

This study demonstrates the improvements in students’ clinical skills achieved with a ward-based course. The implementation of mandatory clinical courses in the national pharmaceutical curriculum is therefore advisable.

## Additional file

Additional file 1: Results of the OSCE. Spreadsheet showing the results of the OSCE assessment (raw data). (XLSX 16 kb)

## Abbreviations

DRP: Drug-related problem
IQR: Interquartile ranges
MAI: Medication Appropriateness Index
OSCE: Objective structured clinical examination
SOAP: subjective, objective, assessment, plan
